# Rare giant maxillay mucocele: A rare case report and literature review

**DOI:** 10.1016/j.amsu.2019.05.013

**Published:** 2019-06-01

**Authors:** Sudha Shahi, Anuj Devkota, Tika Ram Bhandari, Tridip Pantha, Dipendra Gautam

**Affiliations:** a Department of Otorhinolaryngology Head and Neck Surgery, National Academy of Medical Sciences, Bir Hospital, Kathmandu, Nepal; b Department of General Surgery, People's Dental College and Hospital, Kathmandu, Nepal

**Keywords:** Mucocele, Maxillary sinus, Cald well Luc

## Abstract

**Introduction:**

Mucocele is a slow growing, benign but locally aggressive cystic structure lined by true epithelium. It often results due to obstructed sinus outflow or obstruction of gland-like mucous retention cyst. It can cause bony destruction and might result in orbital symptoms like diplopia, orbital displacement, visual disturbances. Other clinical features are facial numbness, dental problems, etc. Radiological evaluation is the preferred diagnostic modality. Surgical removal is the treatment of choice both endoscopic and open (could well luc) approach or combined approach are preferred. Here we report a very typical case of maxillary mucocele who presented with subtle symptoms of nasal obstruction. The study was done in compliance with SCARE guidelines.[1]

**Case presentation:**

We present a very unique case of 24 years man with complaints of nasal obstruction and swelling over the right cheek for 2 years. He had a history of facial trauma two years back. Diagnosis was made on the basis of radiological examination CT (Computed Tomography) scan. He underwent enucleation via Cold well Luc's approach with good postoperative results.

**Conclusion:**

Maxillary mucoceles are slow growing benign lesions. However, they are locally aggressive and cause bony destruction resulting into orbital and dental symptoms. Thus early recognistion with regular folllowr up and planning for surgical intervention can help avoid complications.

## Highlights

• Maxillary sinus mucoceles are rarer entities as compared frontoethmoidal mucoceles.
• We report a case of a giant maxillay sinus mucocele with a history of nasal obstruction and right cheek swelling for 2 years.
• Early diagnosis and surgical intervention is the mainstay of management in most of the cases.

## 1 Introduction

A mucocele is defined as an epithelial-lined cavity containing mucus. It is often described as mucous retention cyst or cystlike and cystic with true epithelial linings. When present in paranasal sinuses they are lined with respiratory epithelium [2] Although it is slow growing and benign in nature, it is locally aggressive and its growth can produce local bone destruction [3,4].Most mucoceles occur in frontal and ethmoidal sinus followed by maxillary sinus whilst sphenoid sinus mucoceles are the least common ones. Maxillary sinus mucoceles represent 2.7–10% of all mucoceles [5]. The etiology of mucocele remains uncertain. However, chronic inflammation of the sinus mucosa due to infection or trauma has been mentioned as the etiology of mucocele formation [6].The chronic inflammation leads to an obstructed sinus outflow resulting in an accumulation of fluid in a mucoperiosteal-lined cavity [2,4,7].

According to the possible mechanisms postulated by Robert.E. Blasley et al., in 1984, th first mechanism states that a mucocele can arise from an epithelial inclusion within the connective tissue stroma of the sinus lining (similar to an epithelial rest of Malassez). Similarly, the second mechanism states that extravasation of mucus into the connective tissue stroma through a ruptured mucous gland leads to reflecting the sinus lining within itself as the gland continues to secrete mucus. This results in formation of mucocele. The third mechanism involves the blockage of a mucous gland. Glands lying within the connective tissue stroma of the sinus lining, as they expand might reflect the normal sinus lining resulting into a mucocele. The fourth mechanism describes the pooling of the mucous discharge from the mucous glands of the sinus due to blockage of the sinus ostium. This mucocele Is lined by the original sinus membrane and will contain mucus. Mechanisms 2 and 3 are the most accepted mechanisms and have been described by previous authors to their studies [2,3] The reason behind the locally aggressive nature of the tumor has been described as the increased Osmotic pressure on continued expansion, and osteoclastic activity with bony expansion. Maxillary antral mucoceles are most of the times asymptomatic or found incidentally. Clinically antral mucocoeles can present with a history of chronic nasal obstruction or fullness, headache, painless cheek swelling, facial numbness, diplopia, visual impairment problems enophthalmos, and dental depending on the size and location of the mucocele [[8], [9], [10]].

The most important diagnostic tools are computerized tomography and magnetic resonance imaging where egg shell bony erosion can be seen in a CT scan [5,7] Treatment is surgical excision of the mucocele. Meanwhile the study was done in compliance with SCARE guideines [1].

## 2 Case presentation

**Fig. 1:**
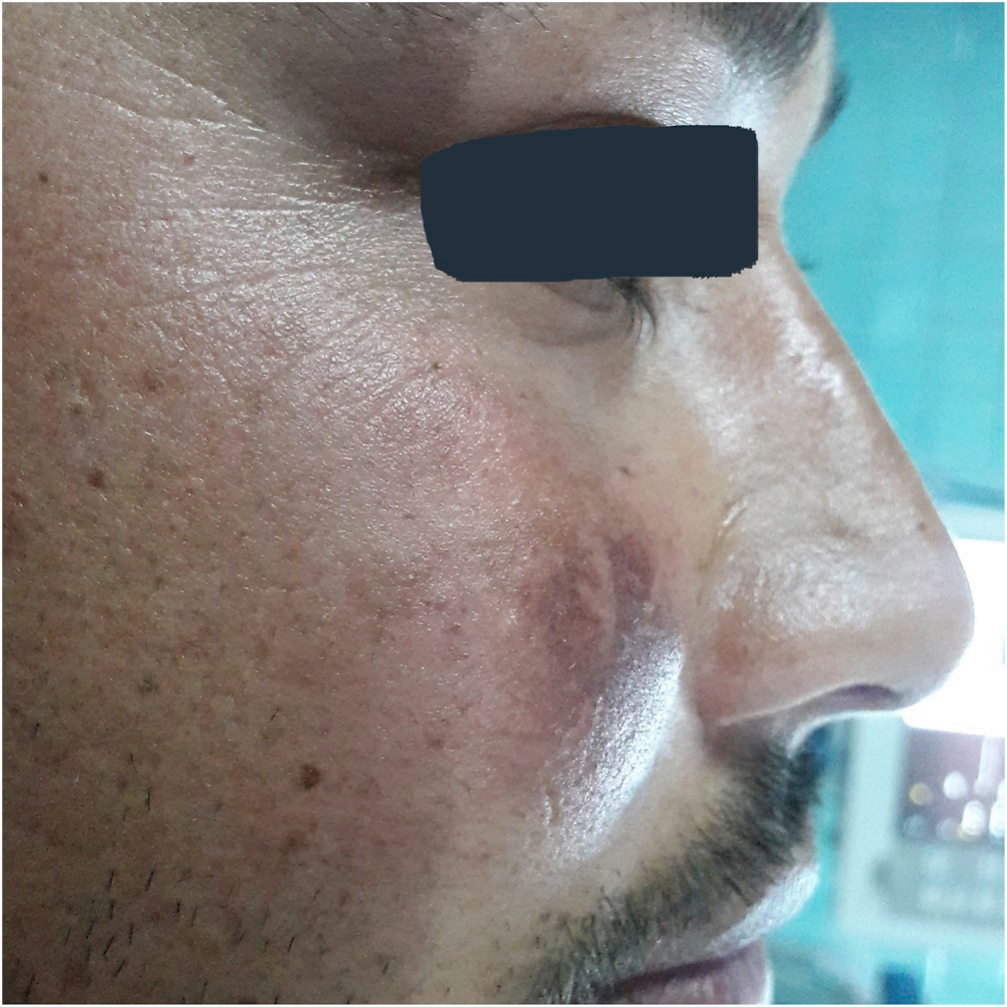
Showing the swelling over right cheek.

**Fig. 2:**
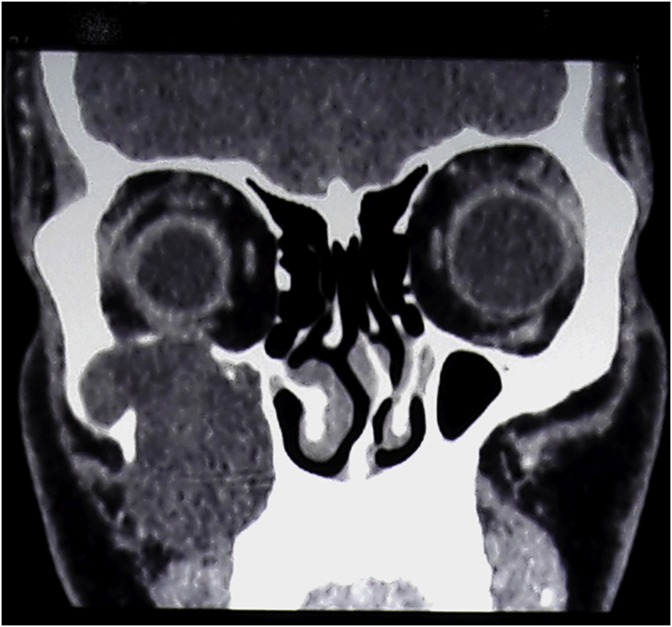
CT showing hypodense lesion in right maxillary sinus.

**Fig. 3:**
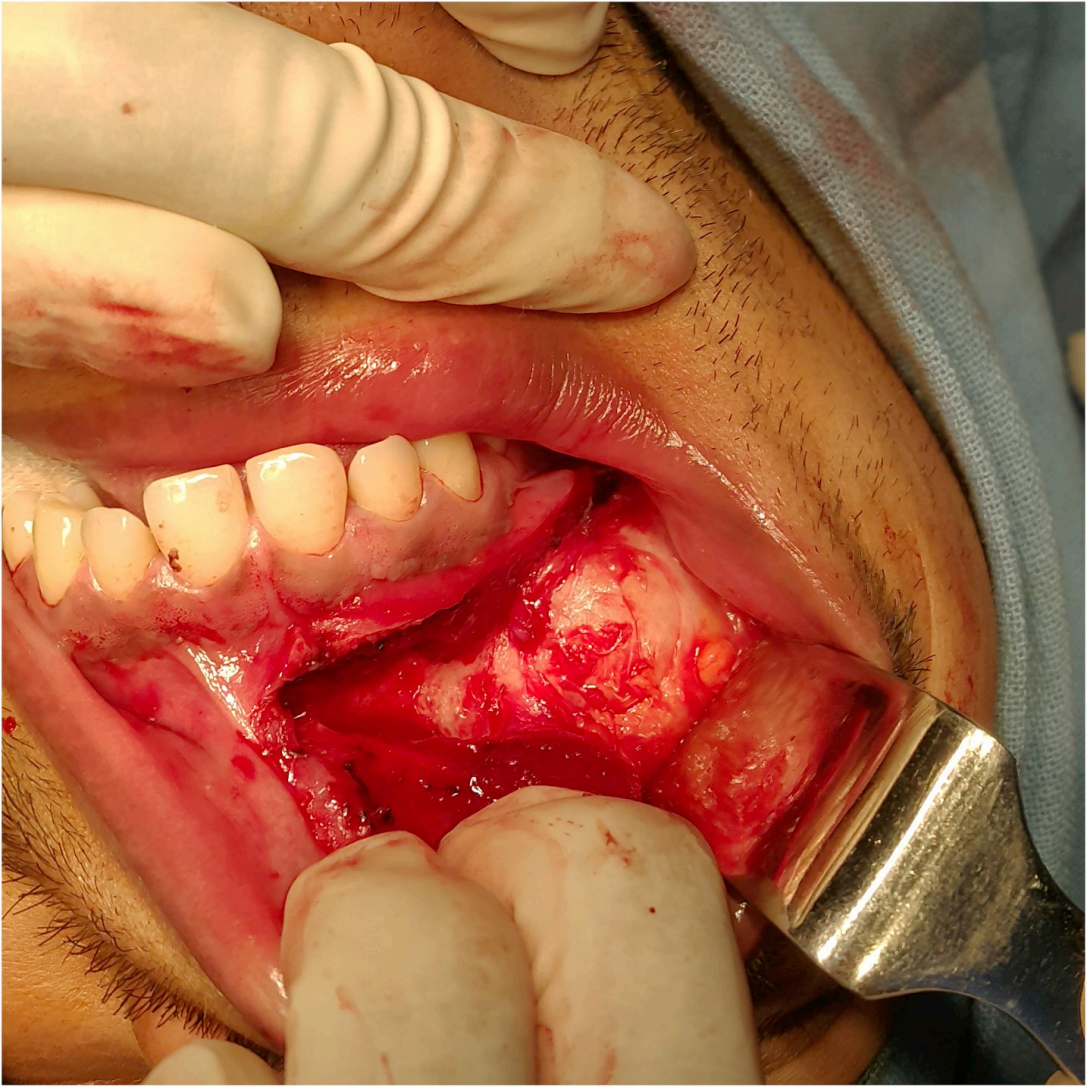
Intraoperative cystic mass.

A 26-year-old man presented with complaints of nasal obstruction for 2 years. He was having nasal obstruction on and off as was on repeated use of topical nasal drops He also complained of an associated headache on and off which was more localized in the right side. He also complained of swelling of the right cheek for the same duration of time. The swelling was insidious in onset and progressively enlarging in size. There was no history of pain, nasal congestion, facial numbness, or any oroantral surgery in the past. However, he gave a history of blunt trauma over his right cheek 5months back. There was no significant family history relevant to the disease. On Inspection, we could see a diffuse swelling of the right cheek with the mild erythematous change of the overlying skin (Fig. 1). On palpation, the swelling was firm, nontender and slightly mobile. Examination of the oral cavity revealed a bulge over the right gingivobuccal sulcus. He was then planned for a CT scan of the paranasal sinus which revealed opacified and expanded right maxillary sinus withlow-density lesion ∼53*44 mm and scalloping and resorption of posteroinferior, medial and superolateral walls and widening of osteomeateal complex (Fig. 2). The features were suggestive of right maxillary mucocele.With the diagnosis above he was planned for Caldwell Luc sinusectomy by a team of Otorhinolaryngology Head and Neck Surgeons under General Anesthesia. Intraoperatively cystic mass (Fig. 3)containing thick mucopurulent content was identified and the around 25 ml of fluid was drained out. All walls of the maxillary sinus appeared thinned out. A large middle meatal antrostomy was performed after exenterating the anterior ethmoidal cells. The histopathological report was consistent with the diagnosis of mucocele.

## 3 Discussion

Mucocele is more common in the frontoethmoidal region and rarely involves the maxillary sinus. The most common etiology for maxillary sinus mucoceles are untreated trauma, history of prior sinus surgery, facial fractures [11]. Antral mucoceles generally involve the lateral sinus wall first, followed by the orbital floor, the hard palate, and less frequently, the medial (nasal) wall [12]. Orbital displacement, proptosis, diplopia, ophthalmoplegia, and decreased visual acuity can result if the superior wall of the maxillary sinus becomes dehiscent [13]. The treatment of a sinus mucocele is surgical. 70–80% cure rated have been reported in different studies [14,15]. The recommended treatment for maxillary sinus mucoceles with no extension to soft tissues of the cheek is endoscopic evacuation with wide middle meatal antrostomy. Traditionally, the recommended treatment is a Caldwell-Luc technique with total removal of the mucocele capsule and wide nasoantral window [12]. But nowadays the traditional method has been replaced by replaced by endoscopic marsupialization with very low recurrence rate at or close to 0% and minimally invasive with a shorter postoperative recovery and less morbidity [16]. However, a Caldwell Luc approach is reserved for more extensive mucocoeles involving facial soft tissues, pterygomaxillary fossa. Similarly, mucoceles developed as a result of facial trauma or previous surgery or those with incomplete enucleation by endoscopic sinus surgery require an open approach.

In our case, we performed enucleation via Caldwell Luc approach. The post operative period was uneventful. He was discharged on the 7th postoperative day. There were no signs of complications or disease recurrence on subsequent follow-up. The patient was compliant with the treatment provided.

## 4 Conclusion

Long-standing benign inflammatory condition of paranasal sinus and blockage of the osteomeatal complex can result in mucocele. Though bening in nature regular follow up and close observation can be helpful in planning the surgical intervention to avoid complications that usually arise due to the locally aggressive nature of the tumor.

## Ethical approval

As this is a case report ethical approval has been exempted and written informed consent has been taken from the patient.

## Sources of funding

No sources of funding

## Author contribution

1-Dr.Sudha Shahi – - Study concept or design, data collection, literature search, writing paper, final decision to publish.

2-Dr.Anuj Devkota-literature search, final decision to publish.

3-Dr.Tika Ram Bhandari- Supervised the writing of the manuscript, final decision to publish.

4.Dr.Tridip Pantha: Supervised the writing of the manuscript, final decision to publish.

5.Dr.Dipendra Gautam: Supervised the writing of the manuscript, final decision to publish.

## Conflicts of interest

No conflict of interest.

## Registration of research studies

Is a case report.

## Guarantor

Dr. Sudha Shahi.

## Consent

Written informed consent was obtained from the patient for publication of this case report and accompanying images.

A copy of the written consent is available for review by the Editor-in-Chief of this journal as required.

## Competing interests

All authors declare that they have no competing interests.

## Peer review and provenance

Not commissioned, externally peer reviewed.
